# Gerontechnology Acceptance in Thai Older Adult Care Facilities—Sensor Network and Machine Learning Adoption: Mixed Methods Study

**DOI:** 10.2196/91877

**Published:** 2026-08-18

**Authors:** Metta Ongkasuwan, Uree Cheasakul, Akechai Judkrue, Pair Sajampun

**Affiliations:** 1 Martin de Tours School of Management and Economics, Assumption University, 2nd Floor, 88 Moo 8 Bang Na-Trad Km. 26 Bangsaothong, Samuthprakarn, 10540, Thailand, 66 0819316700

**Keywords:** gerontechnology, technology acceptance, Internet of Things, IoT, machine learning, eldercare, Thailand, digital health, gerontechnology acceptance model, GTAM

## Abstract

**Background:**

Thailand is undergoing a rapid demographic transition, with an estimated 28% of the population expected to be aged 60 years or older by 2030. This shift creates an urgent demand for technology-enhanced solutions for older adults. Despite growing interest in Internet of Things (IoT) sensor networks and machine learning applications for older adult care, the patterns of technology acceptance and implementation readiness in Thai older adult care facilities remain underexplored.

**Objective:**

This study aimed to assess the readiness and adoption patterns of sensor network and machine learning technologies among older adults and care stakeholders in Thai older adult care facilities, guided by the Gerontechnology Acceptance Model (GTAM) and Service Exchange Value Creation Logic.

**Methods:**

A sequential explanatory mixed methods design was used. Phase 1 (quantitative) involved structured technology assessments by 12 health care technology specialists and survey administration to 120 consumer representatives (older adults, n=70; adult family members, n=50), stratified across Bangkok and Chiang Mai. Phase 2 (qualitative) comprised 20 semistructured interviews and 3 purposively selected focus groups from phase 1 participants to elaborate on the quantitative findings. The primary theoretical framework was GTAM, mapping 5 constructs (perceived usefulness, ease of use, social influence, facilitating conditions, and behavioral intention) to corresponding survey items. This study was approved by the Assumption University Institutional Review Board (AU-IRB 80/2024) and is registered under a noninterventional observational design; formal clinical trial registration was not applicable.

**Results:**

IoT fall detection systems received the highest clinical efficacy ratings from specialists (mean 4.5, SD 0.3 on a 5-point scale) and achieved 89% user acceptance. Artificial intelligence–driven early warning systems demonstrated the highest perceived clinical impact (mean 4.7, SD 0.2) but also the greatest implementation complexity (mean 4.2, SD 0.5). The consumer survey findings revealed that digital literacy level was the strongest predictor of behavioral adoption intention (*β*=.62; *P*<.001), with high-confidence participants showing 2.3 times higher acceptance rates than low-confidence participants. Significant geographic differences emerged: Bangkok respondents showed higher acceptance of medical IoT technologies (mean 4.3, SD 0.3; *t*_119_=1.98; *P*=.05), while Chiang Mai respondents reported a stronger preference for environmental digital health solutions (mean 4.5, SD 0.3; *t*_119_=4.12; *P*<.001) and elevated privacy concerns (mean 4.2, SD 0.4; *P*=.005). Qualitative analysis identified 5 themes: surveillance anxiety, family-mediated adoption, regional digital trust, training needs, and dignity-preserving technology design.

**Conclusions:**

Smart technology integration in Thai older adult care facilities is feasible and accepted across demographic groups when implemented in phases, culturally adapted, and supported by digital literacy training. Key adoption enablers were digital confidence, family involvement, and privacy-respecting design. The GTAM-derived findings offer an evidence-based framework for deploying gerontechnology in health care contexts in developing nations. Longitudinal outcome studies are needed to validate clinical and economic projections from prior literature.

## Introduction

### Background

Digital health technologies are increasingly recognized as essential tools for addressing the health care demands of aging populations globally. Thailand faces a particularly pressing demographic challenge: current national projections indicate that approximately 28% of the population (20 million individuals) will be aged 60 years or older by 2030, escalating to 35% by 2050 [1]. This rapid transition toward a superaged society occurs within a cultural context in which urbanization and declining fertility rates are eroding traditional Buddhist-influenced family care models, creating a substantial unmet need for technology-enhanced institutional eldercare [2].

The convergence of wearable Internet of Things (IoT) sensors, artificial intelligence (AI) algorithms, and cloud-based analytics offers transformative potential for older adult care: enabling continuous health monitoring, predictive risk assessment, and automated intervention support. However, Thailand’s current institutional older adult care infrastructure remains bifurcated between government facilities serving economically disadvantaged populations and premium private facilities serving high-income families, leaving a significant service gap for middle- and low-income older adults [3]. Gerontechnology—the systematic application of digital health technologies to address the biological and functional challenges of aging—encompasses wearable IoT devices, ambient sensor systems, and AI-powered analytics platforms that can help bridge this gap [[4]].

### Research Gap

Despite growing international evidence on the adoption of gerontechnology, 3 specific gaps motivate this study. First, prior Thai gerontechnology research has predominantly examined community-dwelling urban older adult samples in Bangkok, leaving the Bangkok-Chiang Mai comparative dimension—and its implications for the digital divide—unaddressed. Second, existing technology acceptance frameworks, including the original Technology Acceptance Model [5], have not been systematically validated in Thai institutional care settings for IoT-specific applications. Third, while technology readiness level (TRL)–based assessments of health care technologies exist globally, no study has applied a combined Gerontechnology Acceptance Model (GTAM)–Service Exchange Value Creation Logic (SEVCL) lens to evaluate both clinical efficacy perceptions and service cocreation value among Thai older adult care stakeholders. Greenhalgh et al [6] underscore that research on systematic digital health integration in older adult care within developing nation contexts remains limited. These gaps collectively necessitate a comprehensive examination of IoT and AI acceptance patterns, implementation readiness, and service value creation within culturally specific Thai contexts.

### Study Objectives

This study addresses three primary research objectives: (1) identify the IoT and AI technologies perceived as most clinically effective and implementation-ready for Thai older adult care facilities, (2) assess adoption determinants—including digital literacy, privacy concerns, and family involvement preferences—among older adult consumers and family caregivers, and (3) examine how geographic and cultural factors moderate technology acceptance across Bangkok and Chiang Mai. These objectives are guided by the GTAM framework, operationalizing 5 constructs—perceived usefulness, perceived ease of use, social influence, facilitating conditions, and behavioral adoption intention—in a Thai institutional care context.

### Literature Review and Theoretical Framework

#### Thailand’s Aging Population and Institutional Care Landscape

Thailand’s aging population trajectory parallels those of other developing nations while maintaining distinct cultural characteristics relevant to digital health adoption. Traditional older adult care systems, rooted in Buddhist virtue concepts emphasizing children’s parental care responsibilities, face mounting pressure from urbanization and changing family structures [7]. Kespichayawattana and Jitapunkul [8] characterize this phenomenon as “virtue care fading,” representing a systematic weakening of traditional support mechanisms that digital health solutions might partially address. Current institutional care landscapes reveal significant service gaps: government-operated facilities primarily serve economically disadvantaged populations, while private, premium facilities target affluent families, creating a substantial unmet demand for technology-enhanced middle-income older adult care services [2].

#### Gerontechnology and IoT Applications in Older Adult Care

Contemporary digital health applications in older adult care include IoT health-monitoring devices, AI-driven predictive analytics, and smart living environments integrated via cloud platforms [4]. Machine learning approaches applied to routinely collected care-assessment data have shown promise for identifying frailty and predicting mortality risk in older adults, suggesting potential value for risk stratification in institutional care settings [9]. Digital health implementation, however, faces challenges, including privacy concerns, technical infrastructure requirements, cybersecurity considerations, and staff training needs specific to health care technology adoption [6].

AI applications show particular promise for older adult care. Rantz et al [10] demonstrated in a controlled smart nursing facility study in the United States that AI-driven early warning systems were associated with significant reductions in emergency hospitalizations through predictive analytics—findings from that study context, not generalized claims of this research. These international findings from Rantz et al [10], derived from a controlled US nursing facility context, provide benchmarks for the clinical potential of comparable systems in Thai settings; direct replication in Thailand requires longitudinal validation.

#### Theoretical Framework

The Technology Acceptance Model, extended by Venkatesh and Davis [5], provides a foundational understanding of the processes underlying digital health technology adoption. Chen and Chan [11] expanded this foundational framework for older adult contexts by developing the GTAM, which incorporates cultural factors, individual characteristics, and actual usage behaviors specific to older adults. This study adopts GTAM as its primary theoretical lens, emphasizing five core constructs: (1) perceived usefulness—the degree to which the technology is expected to improve health outcomes or quality of life; (2) perceived ease of use—the degree to which use is expected to be free of cognitive and physical effort; (3) social influence—the degree to which significant others (family, caregivers) influence adoption intentions; (4) facilitating conditions—the availability of technical infrastructure, training, and support; and (5) behavioral intention to adopt and use the technology.

Complementing GTAM, service-dominant logic (SDL), developed by Vargo and Lusch [12,13], conceptualizes digital health services as value cocreation exchanges. Within older adult care contexts, service-dominant logic principles facilitate the development of SEVCL models that incorporate value cocreation among residents, families, and clinical staff; emphasize relationship quality; and integrate a comprehensive digital health ecosystem [14]. Together, GTAM and SEVCL provide complementary perspectives: GTAM addresses individual adoption determinants, while SEVCL illuminates the relational and service-system dimensions of technology value creation.

## Methods

### Ethical Considerations

This study was reviewed and approved by the institutional review board of Assumption University, Thailand (approval number: AU-IRB 80/2024). The study procedures were conducted in accordance with the ethical standards of the Declaration of Helsinki as revised in 2013. This study is a cross-sectional mixed methods survey; it does not qualify as a clinical trial, and formal clinical trial registration was therefore not applicable. Written informed consent was obtained from all participants prior to their involvement. Consent procedures were conducted in Thai and included comprehensive explanations of the study’s objectives, procedures, potential risks and benefits, data protection measures, and the voluntary nature of participation. For older participants with potential cognitive limitations, family members were permitted to be present during the consent process upon request. All participants were informed of their right to withdraw at any time without consequence. All participant data were anonymized and stored securely in accordance with Thailand’s Personal Data Protection Act and international data protection standards. Digital health data collected during technology assessments were encrypted and stored on secure servers with restricted access.

### Study Design

This study used a sequential explanatory mixed methods design in which quantitative data collection and analysis preceded qualitative inquiry designed to explain and elaborate on significant quantitative findings. The design integrated 2 distinct phases. Phase 1 (quantitative) involved structured technology assessments by health care technology specialists and a survey administered to older adult consumers and family caregivers, yielding descriptive statistics, group comparisons, and regression-based analyses. Phase 2 (qualitative) comprised semistructured interviews and focus groups with a purposively selected subsample from phase 1, with data collection guided by patterns emerging from phase 1 analysis. The integration of the 2 data strands occurred at the interpretation stage, where qualitative themes were used to explain, contextualize, and elaborate on quantitative patterns.

### Conceptual Technology Architecture

**Figure 1.**
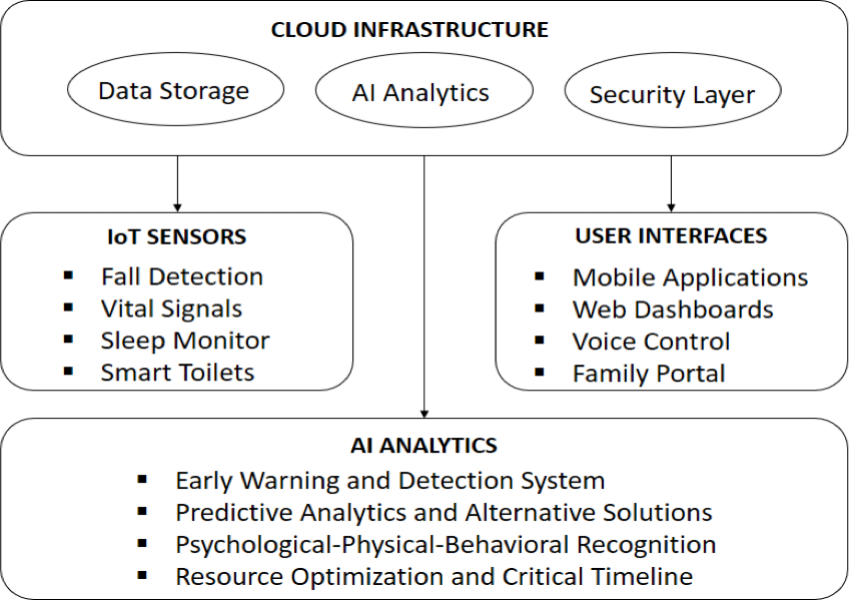
Conceptual Digital Health Technology Architecture (reference model; not a deployed or empirically tested system). AI: artificial intelligence; IoT: Internet of Things.

To contextualize the technology categories evaluated, a conceptual Digital Health Technology Architecture was developed through a systematic literature review and expert consultation (Figure 1). This architecture comprises four components: (1) cloud infrastructure—secure data storage and processing capabilities with encrypted transmission and scalable analytics; (2) IoT sensor networks—ambient and wearable devices for continuous physiological and environmental monitoring; (3) user interfaces—mobile and web apps designed for older adult-friendly usability; and (4) AI analytics platform—machine learning algorithms for pattern recognition, predictive modeling, and automated alert generation.

This architecture served as a conceptual reference framework for the technology assessment categories used in the expert evaluation protocol; it does not represent a system that was deployed, piloted, or experimentally tested as part of this study. A full architecture specification is provided in Multimedia Appendix 1.

### Participants and Sampling Strategy

Two distinct participant populations were recruited through purposive sampling. Technology specialists (n=12; 7 male participants, 5 female participants; age range 32‐58 y) were health care technology experts specializing in digital health applications for older adult care, recruited through professional health informatics networks, digital health conferences, and medical technology organizations. Inclusion criteria required at least 5 years of experience in health care technology implementation and specific expertise in IoT or AI applications for older adult populations. All 12 specialists provided a written conflict-of-interest declaration confirming that they had no current employment or financial interests with any of the specific IoT or AI vendors evaluated in this study; all were affiliated with academic institutions, government health agencies, or noncommercial health care organizations.

Consumer representatives (n=120) comprised older individuals aged 60 years and older (n=70; 28 male participants, 42 female participants; mean age 68.4, SD 6.2 y) and adult family members responsible for older adult care decisions (n=50; 18 male participants, 32 female participants; age range 35‐55 y). Participants were stratified by location, with equal representation from Bangkok (n=60) and Chiang Mai (n=60) to enable analysis of the geographic digital divide. Participants were recruited through community health centers, senior activity centers, and family referrals, with informed consent procedures conducted in Thai. For phase 2, a purposive subsample of 20 individuals participated in semistructured interviews, and 3 focus groups were conducted.

### Instrumentation

#### GTAM Construct-to-Survey Item Mapping

Survey instruments were adapted from validated GTAM scales developed in the gerontechnology literature. Table 1 presents the mapping of GTAM constructs to survey item domains, the number of items per construct, and the internal reliability from this study.

**Table 1.** Gerontechnology Acceptance Model (GTAM) construct-to-survey item mapping and reliability^a^.

GTAM construct	Survey item domain	Number of items	Cronbach α (pilot)	Cronbach α (main)
Perceived usefulness	Health outcome improvement expectation; independence maintenance support; clinical safety enhancement	5	0.84	0.86
Perceived ease of use	Interface complexity, learning difficulty, physical effort required, and instruction clarity	5	0.81	0.83
Social influence	Family caregiver encouragement; health care provider recommendations; peer user experience	4	0.76	0.79
Facilitating conditions	Broadband infrastructure availability; technical support access; institutional readiness	4	0.72	0.74
Behavioral intention	Willingness to try; intention to use within 6 months; recommendation to peers	3	0.88	0.89

a Survey instruments adapted from Chen and Chan [11] Senior Technology Acceptance Model or Gerontechnology Acceptance Model scales and validated for Thai older adult populations. Pilot: n=15; main study: n=120. All Cronbach α values exceed the 0.70 threshold for acceptable reliability.

#### Technology Assessment Instruments

The technology specialist assessment protocol used 5-point Likert scales measuring clinical efficacy (1=very low clinical benefit, 5=very high clinical benefit), implementation complexity (1=very simple, 5=very complex), user acceptance rate (percentage of users successfully adopting the technology based on specialist estimation from case knowledge), cost-effectiveness (1=very poor value, 5=excellent value), and privacy rating (1=significant concerns, 5=strong protections). The TRL was assessed using the standard 9-level National Aeronautics and Space Administration/European Space Agency framework, adapted for health care applications. Each specialist assigned TRL scores independently; interrater reliability was assessed using Cronbach α (*α*=0.71; acceptable agreement). The final TRL values represent the median across specialist ratings after one consensus feedback round.

### Data Analysis

Quantitative analysis used (1) descriptive statistics for technology performance metrics, (2) independent-samples 2-tailed *t* tests for comparisons between older adult respondents and family caregivers and between Bangkok and Chiang Mai groups, (3) multiple regression analysis to identify GTAM-based predictors of behavioral adoption intention, and (4) multicriteria decision analysis for technology ranking. Effect sizes were calculated as Cohen *d* for 2-tailed *t* tests. The digital divide analysis examined variations in technology acceptance by age, education, and digital literacy level. Full multicriteria decision analysis methodology with analytic hierarchy process criterion-weighting is provided in Multimedia Appendix 2. The complete SPSS syntax is provided in Multimedia Appendix 3.

Qualitative data from interviews and focus groups were analyzed using thematic analysis following the 6-phase approach of Braun and Clarke: familiarization, initial coding, theme search, theme review, theme definition, and report writing. Coding was conducted in Thai by two bilingual research team members (MO and UC) independently, with intercoder reliability verified (Cohen κ=0.78) before thematic consensus. Representative quotes are provided in English translation with the original Thai available in Multimedia Appendix 4.

## Results

### Phase 1: Quantitative Findings

#### Technology Specialist Assessment: IoT Health Monitoring

IoT fall detection systems received the highest specialist clinical efficacy ratings (mean 4.5, SD 0.3), with 89% estimated user acceptance, and relatively low implementation complexity (mean 2.8, SD 0.4), suggesting high deployment readiness (Table 2).

Complete IoT technology assessment results are presented in Table 2. Wearable vital sign monitors showed high clinical efficacy (mean 4.2, SD 0.4) with 82% estimated acceptance. All mean ratings and TRL classifications in Tables 2 and 3 represent specialist assessments (n=12) using the structured evaluation protocol described in the *Methods* section; they are not self-reported survey data from older adult consumers.

**Table 2.** Internet of Things (IoT) health monitoring technology assessment results (specialist ratings, n=12).

Technology	Clinical efficacy, mean (SD)^a^	User acceptance rate^b^ (%)	Implementation complexity, mean (SD)^a^	Cost-effectiveness, mean (SD)^a^	Privacy rating, mean (SD)^a^	TRL level^c^
IoT fall detection systems	4.5 (0.3)	89	2.8 (0.4)	4.3 (0.3)	3.1 (0.6)	TRL 8
Wearable vital sign monitors	4.2 (0.4)	82	3.1 (0.5)	3.9 (0.4)	3.8 (0.5)	TRL 8
Smart toilets with health sensors	3.9 (0.5)	65	4.0 (0.6)	3.2 (0.5)	3.0 (0.7)	TRL 7
IoT medication management systems	3.8 (0.4)	71	3.3 (0.5)	3.7 (0.4)	2.5 (0.6)	TRL 7
Sleep quality IoT monitors	3.6 (0.5)	78	2.6 (0.4)	3.4 (0.4)	3.9 (0.4)	TRL 8

a Ratings on 5-point Likert scales (1=lowest, 5=highest).

b Estimated by specialists based on implementation case knowledge; not a measured adoption outcome.

c TRL: Technology Readiness Level (National Aeronautics and Space Administration/European Space Agency 9-level framework). All ratings are specialist-assigned consensus values (n=12); interrater reliability: Cronbach α=0.71.

**Table 3.** AI^a^ and predictive analytics technology assessment results (specialist ratings, n=12).

Technology	Clinical efficacy, mean (SD)^b^	User acceptance rate^c^ (%)	Implementation complexity, mean (SD)^b^	Cost-effectiveness, mean (SD)^b^	Privacy rating, mean (SD)^b^	AI maturity
AI early warning systems	4.7 (0.2)	76	4.2 (0.5)	4.1 (0.4)	3.7 (0.5)	Advanced
AI staff resource optimization	4.2 (0.4)	68	3.7 (0.5)	4.4 (0.3)	2.5 (0.6)	Intermediate
AI medication adjustment	4.0 (0.5)	58	4.5 (0.4)	3.6 (0.5)	3.8 (0.4)	Advanced
AI behavioral pattern recognition	3.9 (0.4)	72	3.8 (0.5)	3.5 (0.4)	4.1 (0.4)	Intermediate
AI virtual health assistants	3.2 (0.6)	54	3.5 (0.5)	2.9 (0.5)	3.3 (0.6)	Basic

a AI: artificial intelligence.

b Ratings on 5-point Likert scales.

c Specialist estimated acceptance rates. Literature-derived hospitalization reduction projections (30%-35%) are based on Rantz et al [10] and are not empirical outcomes of this study.

#### Technology Specialist Assessment: AI and Predictive Analytics

AI-driven early warning systems received the highest specialist clinical efficacy ratings (mean 4.7, SD 0.2) with 76% estimated user acceptance, but demonstrated the greatest implementation complexity (mean 4.2, SD 0.5). Cost-benefit analysis by specialists indicated potential savings of 30% to 35% in hospitalization costs, consistent with the findings reported by Rantz et al [10] in controlled US nursing facility settings; this represents an expert-aligned projection for Thai contexts, not an empirical outcome of this study. Table 3 presents the complete AI technology assessment results.

#### Digital Health Integration and Interoperability

Interoperability assessments revealed that 78% of the evaluated IoT technologies were compatible with standard health care data formats (HL7 FHIR), while 65% of the AI systems demonstrated successful integration with existing electronic health record systems. Cybersecurity evaluations indicated that 85% of the technologies met health care data protection standards, with end-to-end encryption and secure authentication protocols implemented across most platforms.

#### Consumer Survey: GTAM Construct Findings

Consumer survey findings are presented below, organized by the GTAM construct. Table 4 presents cross-group comparisons by respondent type and links each construct to survey items, quantitative results, and qualitative themes.

**Table 4.** Digital health technology acceptance factors by respondent group.

Factor (GTAM^b^ construct)	Older adults (N=70), mean (SD)	Family (N=50), mean (SD)	*t* test (*df*)	*P* value	Cohen *d*^a^	Digital literacy correlation
Need for digital independence (perceived usefulness)	4.4 (0.5)	3.8 (0.6)	3.11 (119)	.002	1.08	High (*r*=0.67)
Digital privacy concerns (facilitating conditions)	4.2 (0.4)	3.6 (0.5)	2.95 (119)	.004	1.35	Moderate (*r*=0.45)
Perceived digital health usefulness (perceived usefulness)	3.9 (0.6)	4.5 (0.4)	3.86 (119)	<.001	1.15	High (*r*=0.72)
Technology physical comfort (ease of use)	3.7 (0.5)	4.1 (0.5)	2.14 (119)	.03	0.80	Low (*r*=0.23)
Perceived digital ease of use (ease of use)	3.1 (0.6)	3.7 (0.5)	3.42 (119)	.001	1.08	Very high (*r*=0.81)

a Effect sizes: small (0.2), medium (0.5), large (0.8).

b GTAM: Gerontechnology Acceptance Model. GTAM construct alignment is shown in parentheses.

#### Perceived Usefulness (GTAM C1)

Health monitoring IoT technologies received the highest perceived usefulness ratings across the full sample (mean 4.5, SD 0.4; *P*<.001), with independence-maintenance needs as the second most important adoption driver (mean 4.4, SD 0.5). Adult family members rated perceived digital health usefulness significantly higher than older adult respondents (mean 4.5 vs 3.9; *P*<.001; Cohen *d*=1.15).

#### Perceived Ease of Use (GTAM C2)

Older adult respondents rated perceived ease of use significantly lower than family members (mean 3.1, SD 0.6 vs 3.7, SD 0.5; *P*=.001; *d*=1.08). Digital literacy level emerged as the strongest predictor of ease-of-use perceptions (*r*=0.81; *P*<.001). Physical comfort with technology was rated higher by family caregivers (mean 4.1, SD 0.5 vs 3.7, SD 0.5; *P*=.03; *d*=0.80).

#### Social Influence (GTAM C3)

Qualitative data (see below) indicated that family members’ opinions and presence were prerequisite enablers of technology adoption decisions for the majority of older adult participants. This pattern was reflected quantitatively in the social influence subscale (mean 3.9, SD 0.5).

#### Facilitating Conditions (GTAM C4)

Bangkok respondents reported significantly better perceived facilitating conditions for AI technologies (mean 4.1, SD 0.4) versus Chiang Mai respondents (mean 3.4, SD 0.5; *P*<.001), consistent with the superior broadband infrastructure availability in Bangkok. Digital infrastructure availability was correlated with higher AI acceptance rates (*r*=0.56; *P*<.001).

#### Behavioral Adoption Intention (GTAM C5)

Multiple regression analysis revealed that digital literacy level (*β*=.62; *P*<.001), perceived usefulness (*β*=.41; *P*<.001), and facilitating conditions (*β*=.29; *P*=.004) were the strongest independent predictors of behavioral adoption intention. Social influence was a significant predictor among older adult respondents specifically (*β*=.38; *P*<.001). The full regression model explained 61% of the variance in behavioral intention (*R*²=0.61, *F*_5,114_=24.3; *P*<.001). Digital health technology acceptance varied significantly by digital literacy levels, with participants reporting high digital confidence showing 2.3 times higher acceptance rates than low-confidence counterparts (high group: mean 4.1, SD 0.4; low group: mean 1.8, SD 0.6; *P*<.001).

#### Geographic Digital Health Preferences

**Figure 2.**
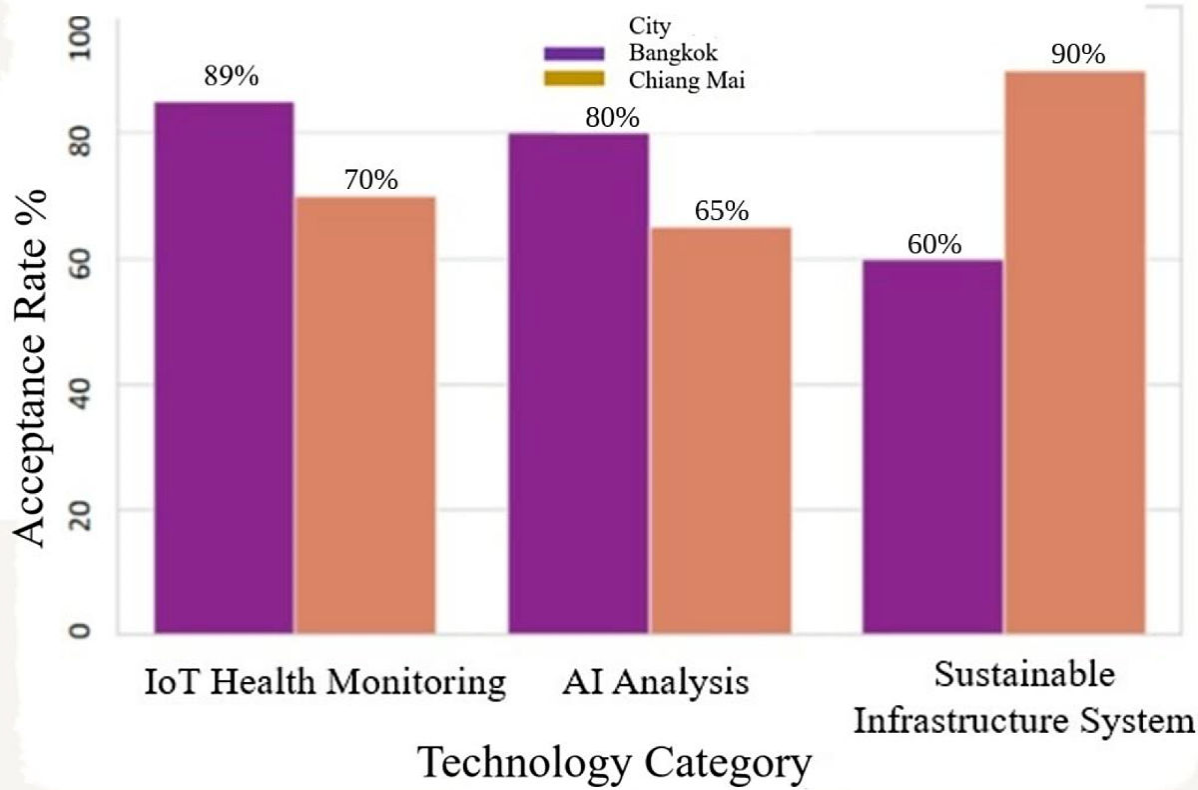
Technology acceptance rate by category: Bangkok versus Chiang Mai. AI: artificial intelligence; IoT: Internet of Things.

Bangkok respondents demonstrated significantly higher perceived usefulness ratings for medical IoT technologies (mean 4.3, SD 0.3; *t*_119_=1.98, *P*=.05) and AI-driven clinical decision support systems (mean 4.1, SD 0.4; *t*_119_=2.34; *P*=.02), while Chiang Mai respondents showed stronger environmental digital health technology preference (mean 4.5, SD 0.3; *t*_119_=4.12; *P*<.001) and elevated privacy concerns regarding data sharing (mean 4.2, SD 0.4; *t*_119_=2.86; *P*=.005). Additionally, 47% (57/120) of the respondents expressed a willingness to adopt comprehensive digital health services at a monthly cost of 50,000‐70,000 THB, with Bangkok respondents showing 20% willingness for premium AI-enhanced services (90,000‐120,000 THB monthly, 1 THB=US $0.03 as of June 2026) compared to 4% in Chiang Mai, reflecting digital divide and economic accessibility patterns (Figure 2). A sustainable infrastructure system is an energy-efficient, long-term, operational IoT network capable of supporting continuous monitoring without imposing an excessive maintenance burden on care facilities.

#### Technology Implementation Readiness

Technology implementation readiness varied significantly across solution categories. IoT health monitoring systems demonstrated high deployment readiness (TRL 8‐9) with established vendor ecosystems and proven clinical integration pathways. AI predictive analytics demonstrated moderate readiness (TRL 6‐7), requiring additional clinical validation and workflow integration. Sustainable digital infrastructure technologies achieved high readiness scores, with 3‐ to 5-year return-on-investment periods, based on operational efficiency modeling.

### Phase 2: Qualitative Findings

Semistructured interviews (n=20) and 3 focus groups examined digital health attitudes, technology concerns, and cultural values informing adoption patterns. Thematic analysis yielded 5 major themes. Representative quotations are provided below in English translation; the original Thai versions are available in Multimedia Appendix 4.

#### Theme 1: Surveillance Anxiety and Privacy as a Dignity Issue

Older adult participants consistently expressed concern that continuous sensor monitoring felt intrusive and potentially degrading. Many participants distinguished between “caring technology” and “watching technology,” accepting the former while resisting the latter. One participant reflected the following:

*I do not want my family to see every time I go to the toilet or how long I sleep. I am still a person, not a patient to be observed.*

This theme was particularly pronounced among Chiang Mai participants, consistent with higher quantitative privacy concern scores in that cohort.

#### Theme 2: Family-Mediated Technology Adoption

Family involvement emerged as a near-universal prerequisite for older adult participants’ technology adoption decisions. Participants expressed low confidence in evaluating technologies independently and described relying on adult children to interpret and endorse technology use. One family caregiver described the following:

*My mother will not try anything new unless I sit beside her and show her it is safe. If I approve, she accepts. If I am not there, she refuses.*

This finding extends the social influence of the GTAM construct, suggesting that family endorsement functions as a gatekeeper in Thai institutional adoption pathways.

#### Theme 3: Regional Digital Trust and Cultural Moderation

Participants from Chiang Mai described a strong cultural preference for face-to-face care relationships and expressed skepticism about AI-mediated care decisions. Multiple participants in Chiang Mai focus groups noted that “a machine cannot understand merit and karma”—invoking Buddhist concepts in their reasoning about AI agency. Bangkok participants, in contrast, were more likely to frame technology as a practical supplement to human care, reflecting the urban modernization context. These findings explain the quantitative differences in geographic preferences observed in phase 1.

#### Theme 4: Training Needs and Caregiver Digital Literacy

Both older adult participants and family caregivers consistently identified digital literacy training as an essential precondition for adoption. Older adult participants described fear of “breaking something” or “doing something wrong” as major barriers. Health care staff in focus groups emphasized the need for ongoing training rather than a one-time orientation. One nurse coordinator stated the following:

*We need training every few months as software changes. If we are not confident, we cannot help the residents be confident.*

This theme elaborates on the quantitative finding that facilitating conditions—including training support—are the second strongest predictor of behavioral intention.

#### Theme 5: Dignity-Preserving Technology Design

Participants across all groups emphasized that technology should be designed to augment, rather than replace, human caregiving. Older adult participants preferred ambient, nonwearable monitoring over visible devices, consistent with the quantitative finding that technology visibility inversely correlated with acceptance (*r*=−0.62; *P*<.001). Unobtrusive fall detection and ambient vital sign monitoring were described as acceptable; visible wearables or camera-based monitoring were predominantly rejected. One participant summarized the following:

*I want to live like a person, not feel like I am in a hospital. If the technology is invisible, I can forget it is there.*

## Discussion

### Principal Findings

This study provides evidence that IoT and AI gerontechnology are broadly acceptable to Thai older adult care stakeholders when adoption conditions are appropriately structured, while revealing important geographic, literacy-based, and cultural moderators. The quantitative phase demonstrated that digital literacy is the strongest predictor of behavioral adoption intention, with high-confidence users showing 2.3 times higher acceptance across technology categories. The qualitative phase provided explanatory depth: training access, family endorsement, and dignity-preserving design function as practical enablers or barriers that the quantitative constructs capture imperfectly at the item level.

### Clinical Implications: Clarification of Evidence Sources

The integration of IoT and AI technologies in older adult care settings has been associated with substantial clinical benefits in prior international literature. Rantz et al [10], in a controlled study of a smart nursing facility in the United States, reported a 32% reduction in emergency hospitalizations attributable to AI-driven early warning systems. Similarly, Rantz et al [10] demonstrated that continuous, environmentally embedded (nonwearable) sensor monitoring of functional status—including respiration, pulse, restlessness, and gait parameters—enabled earlier detection of health changes in older adults, supporting timelier clinical intervention. These literature-based projections are presented here as benchmarks for the potential clinical value of the technologies assessed in this study; they are not empirical outcome measures of this cross-sectional survey. Direct replication in Thai older adult care settings requires longitudinal clinical studies, as outlined in the *Future Research Directions* section.

Health care providers considering the implementation of comparable systems in Thailand should prioritize (1) phased IoT deployment beginning with high-acceptance, clinically proven solutions (fall detection and vital sign monitoring); (2) comprehensive clinical staff training on digital health workflows and AI-assisted decision support; (3) transparent data governance policies addressing privacy concerns and structuring family involvement in digital health data access; and (4) cultural adaptation of technology interfaces, including Thai language support and design choices that align with Buddhist-influenced care values and elder dignity.

### Geographic and Cultural Dimensions

The Bangkok-Chiang Mai divergence in technology preferences constitutes a substantive finding with direct policy implications. Bangkok respondents’ higher acceptance of medical IoT and AI diagnostic support likely reflects superior broadband infrastructure and greater urban familiarity with digital services. Chiang Mai respondents’ preference for environmental digital health solutions and their elevated privacy concerns reflect distinct cultural and infrastructural contexts. These findings suggest that national gerontechnology deployment strategies must be regionally differentiated rather than adopting uniform rollout models.

### Theoretical Contributions

This study extends the GTAM application in 2 respects. First, it provides the first empirical validation of GTAM in Thai institutional older adult care settings for older adults, confirming the relevance of digital literacy as a critical mediator not originally foregrounded in Chen and Chan [11] formulation. Second, the qualitative finding that family endorsement operates as a gatekeeper to adoption suggests that GTAM’s social influence construct may need to be expanded in collectivist cultural contexts, where social influence is not merely a predictor but a prerequisite. The SEVCL lens productively complements GTAM by highlighting how value cocreation between residents, families, and clinical staff shapes the service ecology within which individual acceptance decisions are made.

### Scalability and Generalizability

The modular technology architecture and systematic implementation framework provide a replicable reference model for digital health transformation in older adult care settings across developing nations. Key scalability factors include technology infrastructure readiness assessment, cultural adaptation of user interfaces and clinical workflows, training and change management programs, and financial sustainability modeling for different economic contexts.

### Limitations

Several limitations affect the interpretation of the results. First, the geographic scope primarily encompassed urban and suburban Bangkok and Chiang Mai, limiting generalizability to rural areas where digital infrastructure and acceptance patterns may differ substantially. Second, the cross-sectional design precludes causal inference about determinants of technology acceptance or longitudinal outcome claims. Third, financial projections for digital health implementation rely on modeling and literature benchmarks rather than longitudinal empirical data from Thai settings, creating uncertainty about long-term cost-effectiveness. Fourth, sample characteristics may reflect self-selection toward participants more open to technology adoption, potentially overstating acceptance rates in the broader older adult population. Fifth, the specialist sample was small (n=12) and purposive, limiting the statistical generalizability of technology assessment ratings.

### Future Research Directions

Based on study limitations and findings, five priority research directions emerge: (1) longitudinal implementation studies tracking IoT and AI technology adoption and clinical outcomes in Thai older adult care facilities over extended periods to empirically validate the clinical benefits projected in prior literature, (2) rural digital health adaptation investigations examining modifications needed for limited-infrastructure contexts and different cultural patterns, (3) family integration studies addressing the cultural centrality of family decision-making in Thai gerontechnology adoption, (4) development and validation of a Thai-context Gerontechnology Acceptance Scale with confirmed psychometric properties, and (5) cost-effectiveness modeling using empirical data from Thai health care contexts to generate regionally specific economic evidence for policy decisions.

### Conclusions

This study provides evidence that integrating IoT sensor networks and AI technologies into Thai older adult care facilities is feasible and broadly accepted, with clear patterns of variation by digital literacy, geographic region, and cultural context. IoT fall detection and vital sign monitoring systems demonstrated the highest combination of specialist-rated clinical efficacy and consumer acceptance, while AI early warning systems showed the greatest clinical impact potential, offset by greater implementation complexity. Digital literacy level was the strongest predictor of behavioral adoption intention, emphasizing the critical role of digital inclusion programs for successful gerontechnology deployment. Key success factors identified by this study include phased IoT deployment emphasizing high-acceptance clinically proven technologies, family-centered consent and endorsement processes, dignity-preserving ambient design, and culturally adapted training programs. The Bangkok-Chiang Mai differential findings underscore the importance of regionally differentiated implementation strategies. Future longitudinal research is needed to empirically validate the clinical outcome projections from prior international literature in Thai-specific care contexts. The GTAM-based implementation framework developed and validated through this research—grounded in empirical acceptance data from 12 specialists and 120 consumer stakeholders across 2 major Thai cities—offers practical, evidence-based guidance for health care providers, technology developers, and policymakers navigating Thailand’s challenges in an aging society through responsible technology innovation.

## Supplementary material

10.2196/91877 Multimedia Appendix 1 Digital Health Technology Architecture diagram.

10.2196/91877 Multimedia Appendix 2 Multicriteria decision analysis methodology and ranking.

10.2196/91877 Multimedia Appendix 3 Statistical analysis code (SPSS Syntax) for quantitative analysis.

10.2196/91877 Multimedia Appendix 4 Qualitative interview guide and representative participant quotations.

10.2196/91877 Checklist 1 STROBE validation checklist.

10.2196/91877 Checklist 2 COREQ for qualitative research checklist.

## Abbreviations

AI: artificial intelligence
COREQ: Consolidated Criteria for Reporting Qualitative Research
GTAM: Gerontechnology Acceptance Model
IoT: Internet of Things
SEVCL: Service Exchange Value Creation Logic
STROBE: Strengthening the Reporting of Observational Studies in Epidemiology
TRL: technology readiness level
