# Supplementary material for: Gerontechnology Acceptance in Thai Older Adult Care Facilities—Sensor Network and Machine Learning Adoption: Mixed Methods Study
Source: JMIR Form Res. 2026 Aug 18;10:e91877. doi: 10.2196/91877 (PMC13484945; doi:10.2196/91877)
Supplement: Supplementary file 1 [file formative-v10-e91877-s001.docx]

**Multimedia Appendix 1**

**Digital Health Technology Architecture (DHTA)**

*Conceptual Reference Framework —* ***Not*** *an Empirically Deployed System*

Manuscript #91877 | JMIR Formative Research | Ongkasuwan M et al.

Appendix 1 provides the full specification of the **D**igital **H**ealth **T**echnology **A**rchitecture (DHTA) conceptual reference model used to organize the technology assessment categories in this study. This architecture, with its *diagram and description*, was developed through a systematic literature review and expert consultation during the study design phase. **DHTA** in this study represents *a proposed integration model* for smart elderly care facilities in Thailand, *not a system that was deployed, piloted, or experimentally tested as part of this research.*

**1. Architecture Overview (DHTA)**

The **D**igital **H**ealth **T**echnology **A**rchitecture **(DHTA)** diagram and description for integrated smart elderly care comprises four interdependent layers: (1) Cloud Infrastructure, (2) IoT Sensor Networks, (3) User Interface Systems, and (4) AI Analytics Platform. See Figure 1.

These layers interact in a hierarchical data-flow arrangement: IoT sensors generate raw physiological and environmental data; cloud infrastructure stores and processes this data; AI analytics platforms extract patterns and generate alerts; and user interfaces present actionable information to residents, families, and clinical staff.

Figure 1. Digital Health Technology Architecture Diagram

**2. Component Specifications for DHTA**

**Layer 1: Cloud Infrastructure**

The cloud infrastructure layer provides the foundational data management and computational resources for the entire system. Key specifications include:

- **Data Storage Architecture:**
- Encrypted relational database for structured health records (**HL7 FHIR**-compliant) (i.e., **HL7**= Health Level 7, and **FHIR** = Fast Healthcare Interoperability Resources)
- Time-series database for continuous IoT sensor data streams
- Redundant backup systems with 99.9% uptime service level agreement
- Scalable cloud computing resources (**AWS / Azure** healthcare-compliant instances) (i.e., offers robust security measures where AWS healthcare & Life Sciences for AI and big data analytic services and digital imaging communication in medicine (**DICOM**); and Azure healthcare for Microsoft ecosystem integration on **PaaS**-Platform as a service tool in the cloud)
- Data sovereignty compliance: data storage within Thailand as per **PDPA** (Personal Data Protection Act) requirements
- **Security Protocols:**
- End-to-end **AES-256** (i.e., world top-tier security standard using 256-bit key to encrypt data in 128-bit blocks, passing through 14 rounds of transformation) encryption for all data transmission
- Multi-factor authentication for all clinician and family portal access
- Role-based access control (**RBAC**) (i.e., security method based on authorization of users' roles in the organization), separating resident, family, clinical staff, and administrative access layers
- **HIPAA** (Health Insurance Portability and Accountability Act of 1996) and **PDPA**-aligned audit logging for patient data protection. Thai citizens receive free public healthcare through the **UCS** (Universal Coverage Scheme)

**Layer 2: IoT Sensor Networks**

The IoT sensor network layer integrates ambient and wearable monitoring devices to continuously collect physiological and environmental data. Device categories are described below.

| **Device Category** | **Key Sensors** | **Data Collected** | **Connectivity** | **TRL Range** |
| --- | --- | --- | --- | --- |
| Fall Detection | Accelerometers, pressure mats, infrared motion sensors | Movement patterns, fall events, gait analysis | Bluetooth, Zigbee*, LoRaWAN** | TRL 8–9 |
| Vital Sign Monitoring | SpO2 pulse oximeter (Oxygen Saturation), ECG patch (Electrocardiogram Monitor), skin temperature, respiration rate sensor | Heart rate, blood oxygen, body temperature, breathing rate | Bluetooth 5.0, NFC (Near Field Communication) | TRL 8 |
| Smart Toilet Health Sensors | Urine flow sensors, weight sensors, and chemical strip readers | Urinalysis proxies, hydration indicators, and body weight | Embedded Wi-Fi | TRL 7 |
| Sleep Quality Monitoring | Radar/ultrasound non-contact sensors, pressure-sensing mattress overlay | Sleep stages, respiratory rate, and movement | Wi-Fi 6, Bluetooth | TRL 8 |
| Medication Management | Smart pill dispensers, RFID sensors, and camera-based confirmation | Dose timing, adherence, missed doses | Wi-Fi, cellular backup | TRL 7–8 |
| Environmental Monitoring | Temperature, humidity, air quality, and light level sensors | Room conditions, fall risk, and environmental factors | Zigbee mesh network | TRL 9 |

Zigbee* = IEEE 802.15.4 low-power mesh wireless protocol for smart devices with security for home automation

LoRaWAN** = Long Range Wide Area Network, i.e., a low-power, wide-area network protocol over long distances

**Layer 3: User Interface Systems**

User interfaces are designed for three distinct user groups with different usability requirements.

| **User Group** | **Interface Type** | **Key Design Adaptations** |
| --- | --- | --- |
| Elderly Residents | Large-format tablet application; voice-activated interface; simple one-button emergency alert | Minimum font size 18 pt; high-contrast color scheme; simplified 3-step navigation; Thai-language primary; voice control (Thai dialect support); physical panic button fallback |
| Family Caregivers | Mobile application (iOS/Android) and web dashboard | Real-time health status feed; alert notifications; configurable sharing permissions; video call integration with the resident; weekly health summary report.  Contact numbers (i.e., emergency ambulance hotline, police station, fire department, 24-hour security guard, AED hotline) |
| Clinical Staff | Desktop web dashboard; mobile companion app | Multi-resident monitoring grid; priority-ranked alert queue; EHR (Electronic Health Record) integration interface; AI-generated clinical summary; care plan workflow integration |
| Facility Administration | Administrative web portal | Occupancy and resource management; staff allocation dashboard; compliance and audit reporting; financial integration |

**Layer 4: AI Analytics Platform**

The AI analytics layer transforms raw sensor streams into clinically actionable insights using machine learning algorithms.

| **AI Module** | **Algorithm / Approach** | **Clinical Function** | **Maturity Level** |
| --- | --- | --- | --- |
| Early Warning System | XGBoost (for problem learning and decision making); LSTM (Long-Short Term Memory) for time-series, natural language processing, speed recognition | Predicts health deterioration 4–24 hours in advance; triggers tiered alert protocol | Advanced  (TRL 7) |
| Fall Risk Prediction | Random Forest classifier; gait pattern analysis via CNN (Convolutional Neural Networks) | Daily fall risk score; real-time fall event detection and logging | Advanced  (TRL 8) |
| Behavioral Pattern Recognition | Unsupervised clustering (k-means); HMM (Hidden Markov Model) for activity state modeling | Detects deviations from baseline daily activity patterns; depression/anxiety proxies | Intermediate  (TRL 6) |
| Medication Adherence AI | Rule-based scheduling + reinforcement learning for reminders | Automated dispensing reminders; adherence tracking; physician alert for non-compliance | Advanced  (TRL 7) |
| Staff Resource Optimization | Linear programming + demand forecasting ML model | Dynamic staff scheduling based on predicted care load; overtime reduction | Intermediate  (TRL 6) |
| Personalized Care Recommendations | Collaborative filtering + clinical rule engine | Generates individualized care suggestions based on the physician’s health history and peer cohort | Basic  (TRL 5) |

**3. Data Flow Architecture**

Data flow within the system follows a structured pipeline:

(1) IoT sensors continuously stream raw physiological and environmental data via local wireless protocols to an on-premises gateway device;

(2) the gateway applies edge processing for time-sensitive alerts (fall detection response < 2 seconds) before forwarding to cloud storage;

(3) the cloud AI platform processes aggregated data streams to generate predictive models, trend analyses, and non-urgent alerts (latency 1–15 minutes);

(4) processed outputs are pushed to appropriate user interfaces based on RBAC (Role-based Access Control) rules and alert severity protocols; and

(5) All interactions are logged to the audit trail and EHR (Electronic Healthcare Record) integration layer.

**4. Implementation Phases (Conceptual Timeline)**

| **Phase** | **Timeline** | **Components** | **Key Milestones** |
| --- | --- | --- | --- |
| 1 | Months 1–8 | IoT Infrastructure:  cloud setup, Wi-Fi/Zigbee mesh, fall detection sensors, vital sign monitors, user interfaces for staff and families | Cloud infrastructure live;  fall detection operational;  Staff Training Phase 1 |
| 2 | Months 9–14 | Basic Monitoring:  sleep monitors, medication management, family mobile app, basic alert protocols | 80% sensor coverage achieved; family app adopted;  First alert protocol activated |
| 3 | Months 15–20 | AI Integration:  early warning system deployment, behavioral pattern recognition, EHR integration | AI warning system validated against baseline.  EHR integration complete |
| 4 | Months 21–26 | Advanced Analytics:  predictive fall modeling, staff optimization, personalized recommendations | Full AI analytics operational.  staff optimization, reducing overtime costs |
| 5 | Months 27–35 | Full Optimization:  system refinement, outcome measurement, scalability preparation, knowledge transfer | Clinical outcome data collected; ROI assessment;  documentation for replication |

Note. This timeline is a conceptual project plan developed for stakeholder consultation and technology assessment. It does not reflect the actual implementation timelines of the facilities participating in this study.
