# Supplementary material for: Gerontechnology Acceptance in Thai Older Adult Care Facilities—Sensor Network and Machine Learning Adoption: Mixed Methods Study
Source: JMIR Form Res. 2026 Aug 18;10:e91877. doi: 10.2196/91877 (PMC13484945; doi:10.2196/91877)
Supplement: Supplementary file 2 [file formative-v10-e91877-s002.docx]

**Multimedia Appendix 2**

**Multi-Criteria Decision Analysis (MCDA)**

*Technology Ranking Methodology and Full Results Details*

Manuscript #91877 | JMIR Formative Research | Ongkasuwan M et al.

This appendix presents the detailed **M**ulti-**C**riteria **D**ecision **A**nalysis (MCDA) *methodology and Technology Ranking Details* used to rank and compare IoT and AI technologies for priority of implementation and adoption in Thai elderly care facilities.

MCDA was selected because it systematically integrates multiple heterogeneous criteria—clinical, operational, economic, and social—into a single comparative framework, enabling prioritization decisions that cannot be adequately made on single-criterion bases.

**1. MCDA Methodology**

**1.1 Analytical Hierarchy Process (AHP) for Criterion Weighting**

Criterion weights were derived using the **A**nalytical **H**ierarchy **P**rocess (AHP) developed by Saaty (1980). The 12 technology specialists completed pairwise comparison matrices for the six evaluation criteria during the structured assessment session. Pairwise comparisons used Saaty's 1–9 intensity scale (1=equal importance, 9=extreme importance of one criterion over another).

Weights were calculated from the normalized principal eigenvector of the comparison matrix.

The Consistency Ratio (CR) was <0.10 for all matrices, confirming acceptable consistency.

**1.2 Evaluation Criteria and Final Weights (AHP)**

| **#** | **Criterion** | **Definition** | **AHP Weight (%)** | **Rank** |
| --- | --- | --- | --- | --- |
| C1 | Clinical Efficacy | Strength of evidence for improving health outcomes, preventing adverse events, or improving quality of life for elderly residents | 31% | 1 |
| C2 | User Acceptance Rate | Estimated proportion of target users (elderly residents and families) likely to adopt and continue using the technology | 24% | 2 |
| C3 | Implementation Complexity | Resources, expertise, infrastructure, and time required for successful deployment (inverse scale: lower complexity = higher score or greater simplicity) | 18% | 3 |
| C4 | Cost-Effectiveness | Expected value delivered per unit cost over a 3 to 5-year horizon, including capital, operational, and training costs | 14% | 4 |
| C5 | Privacy and Cybersecurity | Adequacy of data protection controls, cybersecurity architecture, and compliance with PDPA and **HL7** (Health Level 7) standards | 8% | 5 |
| C6 | Interoperability | Compatibility with existing **EHR** (Electronic Health Record) systems, **HL7-FHIR** (Fast Healthcare Interoperability Resources) standards, and other technologies in the proposed architecture | 5% | 6 |

**1.3 Scoring Procedure**

Each of the 12 technology specialists independently scored all *technologies* against each criterion using a 5-point Likert scale.

For Implementation Complexity (C3) versus simplicity, raw scores were reverse-coded so that higher final scores indicate more desirable outcomes or greater simplicity.

Mean scores across specialists were calculated for each *technology-criterion combination*. Weighted scores were then computed as: Weighted Score = Σ (Criterion Weight × Mean Criterion Score).

The final IoT and AI technology MCDA rankings were determined by total weighted scores, as follows:

**2. IoT Technology MCDA Rankings**

| **Technology** | **C1 (0.31)** | **C2 (0.24)** | **C3* (0.18)** | **C4 (0.14)** | **C5 (0.08)** | **C6 (0.05)** | **Weighted Score** | **Rank** |
| --- | --- | --- | --- | --- | --- | --- | --- | --- |
| IoT Fall Detection Systems | 4.50 | 3.91 | 4.40 | 4.30 | 3.10 | 4.20 | 4.20 | 1st |
| Wearable Vital Sign Monitors | 4.20 | 3.65 | 4.00 | 3.90 | 3.80 | 4.00 | 3.97 | 2nd |
| IoT Medication Management | 3.80 | 3.25 | 3.90 | 3.70 | 2.50 | 3.60 | 3.56 | 3rd |
| Sleep Quality IoT Monitors | 3.60 | 3.40 | 4.60 | 3.40 | 3.90 | 3.50 | 3.72 | 4th |
| Smart Toilets with Sensors | 3.90 | 2.75 | 3.10 | 3.20 | 3.00 | 3.30 | 3.30 | 5th |

*C3 scores are reverse-coded (original complexity score subtracted from 6), so higher values = greater simplicity of implementation.

**3. AI Technology MCDA Rankings**

| **Technology** | **C1 (0.31)** | **C2 (0.24)** | **C3* (0.18)** | **C4 (0.14)** | **C5 (0.08)** | **C6 (0.05)** | **Weighted Score** | **Rank** |
| --- | --- | --- | --- | --- | --- | --- | --- | --- |
| AI Early Warning Systems | 4.70 | 3.30 | 2.40 | 4.10 | 3.70 | 4.00 | 3.67 | 2nd |
| AI Staff Resource Optimization | 4.20 | 2.90 | 3.40 | 4.40 | 2.50 | 3.80 | 3.60 | 3rd |
| AI Medication Adjustment | 4.00 | 2.60 | 2.20 | 3.60 | 3.80 | 3.40 | 3.25 | 5th |
| AI Behavioral Pattern Recognition | 3.90 | 3.10 | 3.20 | 3.50 | 4.10 | 3.50 | 3.58 | 4th |
| AI Virtual Health Assistants | 3.20 | 2.40 | 3.40 | 2.90 | 3.30 | 3.10 | 2.99 | 1st |

**4. Combined Priority Matrix: Implementation Sequencing**

Integrating MCDA Scores with TRL readiness levels and Consumer Acceptance Data from Phase 1 (in Appendix 3, implementation phases), the following ranking and implementation priority sequence were developed to guide phased deployment and adoption recommendations.

| **Priority** | **Technology** | **MCDA Score** | **Consumer Acceptance** | **TRL** | **Rec. Phase** |
| --- | --- | --- | --- | --- | --- |
| P1 – Immediate | IoT Fall Detection | 4.20 | 89% | TRL 8–9 | Phase 1 |
| P1 – Immediate | Wearable Vital Signs | 3.97 | 82% | TRL 8 | Phase 1 |
| P2 – Near-term | Sleep Quality Monitors | 3.72 | 78% | TRL 8 | Phase 2 |
| P2 – Near-term | AI Early Warning Systems | 3.67 | 76% | TRL 7 | Phase 3 |
| P3 – Medium-term | IoT Medication Management | 3.56 | 71% | TRL 7 | Phase 2 |
| P3 – Medium-term | AI Staff Optimization | 3.60 | 68% | TRL 6 | Phase 4 |
| P3 – Medium-term | AI Behavioral Recognition | 3.58 | 72% | TRL 6 | Phase 4 |
| P4 – Long-term | Smart Toilet Sensors | 3.30 | 65% | TRL 7 | Phase 3+ |
| P4 – Long-term | AI Medication Adjustment | 3.25 | 58% | TRL 7 | Phase 5 |
| P5 – Aspirational | AI Virtual Assistants | 2.99 | 54% | TRL 5 | Phase 5+ |

Note. Saaty TL. The Analytic Hierarchy Process (AHP): Planning, Priority Setting, Resource Allocation. New York: McGraw-Hill, 1980. MCDA approach adapted from: Thokala P, Devlin N, Marsh K, et al. Multiple Criteria Decision Analysis for Health Care Decision Making—an introduction: report 1 of the ISPOR MCDA Emerging Good Practices Task Force. Value Health. 2016;19(1):1-13.
