# Supplementary material for: Gerontechnology Acceptance in Thai Older Adult Care Facilities—Sensor Network and Machine Learning Adoption: Mixed Methods Study
Source: JMIR Form Res. 2026 Aug 18;10:e91877. doi: 10.2196/91877 (PMC13484945; doi:10.2196/91877)
Supplement: Supplementary file 3 [file formative-v10-e91877-s003.docx]

**Multimedia Appendix 3**

**Statistical Analysis Code (SPSS Syntax)**

*Syntax for All Quantitative Analyses Reported in the Manuscript*

Manuscript #91877 | JMIR Formative Research | Ongkasuwan M et al.

The following SPSS (version 28.0, IBM Corp.) syntax code documents all quantitative analyses reported in the revised manuscript. Data file: JMIR91877_MainData.sav.

Variable labels and coding are described in the Data Dictionary (Section 2 of this Appendix).

**Section 1: SPSS Syntax**

```
* ================================================================
* JMIR Formative Research ms#91877
* Ongkasuwan M et al. - Gerontechnology Acceptance Study
* SPSS Syntax - All Reported Analyses
* File: JMIR91877_Syntax_v3.sps
* ================================================================

* ----- SECTION 1: DATA PREPARATION & RELIABILITY -----

* Load and inspect data.
GET FILE='JMIR91877_MainData.sav'.
DATASET NAME MainData WINDOW=FRONT.
FREQUENCIES VARIABLES=ALL /STATISTICS=MEAN STDDEV MINIMUM MAXIMUM.

* Compute GTAM composite scale scores (mean of items).
COMPUTE PU_Score = MEAN(PU1, PU2, PU3, PU4, PU5).
COMPUTE EOU_Score = MEAN(EOU1, EOU2, EOU3, EOU4, EOU5).
COMPUTE SI_Score = MEAN(SI1, SI2, SI3, SI4).
COMPUTE FC_Score = MEAN(FC1, FC2, FC3, FC4).
COMPUTE BI_Score = MEAN(BI1, BI2, BI3).
EXECUTE.

* Cronbach's alpha reliability for each GTAM subscale.
RELIABILITY
  /VARIABLES=PU1 PU2 PU3 PU4 PU5
  /SCALE('Perceived Usefulness') ALL
  /MODEL=ALPHA /STATISTICS=DESCRIPTIVE SCALE /SUMMARY=TOTAL.

RELIABILITY
  /VARIABLES=EOU1 EOU2 EOU3 EOU4 EOU5
  /SCALE('Perceived Ease of Use') ALL
  /MODEL=ALPHA /STATISTICS=DESCRIPTIVE SCALE /SUMMARY=TOTAL.

RELIABILITY
  /VARIABLES=SI1 SI2 SI3 SI4
  /SCALE('Social Influence') ALL
  /MODEL=ALPHA /STATISTICS=DESCRIPTIVE SCALE /SUMMARY=TOTAL.

RELIABILITY
  /VARIABLES=FC1 FC2 FC3 FC4
  /SCALE('Facilitating Conditions') ALL
  /MODEL=ALPHA /STATISTICS=DESCRIPTIVE SCALE /SUMMARY=TOTAL.

RELIABILITY
  /VARIABLES=BI1 BI2 BI3
  /SCALE('Behavioral Intention') ALL
  /MODEL=ALPHA /STATISTICS=DESCRIPTIVE SCALE /SUMMARY=TOTAL.

* ----- SECTION 2: DESCRIPTIVE STATISTICS -----

DESCRIPTIVES VARIABLES=PU_Score EOU_Score SI_Score FC_Score BI_Score
             DigitalLiteracy PrivacyConcern TechComfort
  /STATISTICS=MEAN STDDEV MIN MAX KURTOSIS SKEWNESS.

* Descriptives for technology specialist ratings (n=12).
DESCRIPTIVES VARIABLES=FallDetect_Efficacy FallDetect_Complexity
             FallDetect_Acceptance FallDetect_Cost FallDetect_Privacy
             WearableVS_Efficacy WearableVS_Complexity
             AIEarlyWarn_Efficacy AIEarlyWarn_Complexity
  /STATISTICS=MEAN STDDEV MIN MAX.

* ----- SECTION 3: INDEPENDENT SAMPLES T-TESTS -----
* Compare elderly respondents (Group=1) vs. family caregivers (Group=2).

T-TEST GROUPS=RespondentType(1 2)
  /MISSING=ANALYSIS
  /VARIABLES=PU_Score EOU_Score SI_Score FC_Score BI_Score
              DigitalPrivacyConcern NeedForIndependence TechComfort
  /CRITERIA=CI(.95).

* Effect size (Cohen's d) computed manually or via SPSS PROCESS macro.
* Cohen's d = (M1 - M2) / SDpooled

* Compare Bangkok (City=1) vs. Chiang Mai (City=2).
T-TEST GROUPS=City(1 2)
  /MISSING=ANALYSIS
  /VARIABLES=PU_Score EOU_Score SI_Score FC_Score BI_Score
              MedIoT_Acceptance AIDecSupport_Acceptance
              EnvDigHealth_Acceptance PrivacyConcern
  /CRITERIA=CI(.95).

* ----- SECTION 4: MULTIPLE REGRESSION -----
* DV: Behavioral Intention (BI_Score)
* IVs: GTAM constructs + digital literacy

REGRESSION
  /MISSING LISTWISE
  /STATISTICS COEFF OUTS R ANOVA CHANGE COLLIN TOL ZPP
  /CRITERIA=PIN(.05) POUT(.10)
  /NOORIGIN
  /DEPENDENT BI_Score
  /METHOD=ENTER DigitalLiteracy PU_Score EOU_Score SI_Score FC_Score
  /SCATTERPLOT=(*ZRESID ,*ZPRED)
  /RESIDUALS HISTOGRAM(ZRESID) NORMPROB(ZRESID)
  /SAVE PRED RESID.

* ----- SECTION 5: CORRELATION ANALYSIS -----

CORRELATIONS
  /VARIABLES=BI_Score PU_Score EOU_Score SI_Score FC_Score
              DigitalLiteracy PrivacyConcern TechVisibility
  /PRINT=TWOTAIL SIG
  /MISSING=PAIRWISE.

* ----- SECTION 6: DIGITAL LITERACY SUBGROUP ANALYSIS -----
* High digital confidence vs. low digital confidence comparison.

* Dichotomize digital literacy at median split (median=3.0 on 5-pt scale).
RECODE DigitalLiteracy (1 THRU 3=1)(3.1 THRU 5=2) INTO DigLit_Group.
VALUE LABELS DigLit_Group 1 'Low Confidence' 2 'High Confidence'.
EXECUTE.

MEANS TABLES=BI_Score PU_Score EOU_Score BY DigLit_Group
  /CELLS MEAN STDDEV COUNT.

T-TEST GROUPS=DigLit_Group(1 2)
  /VARIABLES=BI_Score PU_Score EOU_Score
  /CRITERIA=CI(.95).

* ----- SECTION 7: NORMALITY & ASSUMPTION CHECKS -----

EXAMINE VARIABLES=BI_Score PU_Score EOU_Score SI_Score FC_Score
  /PLOT BOXPLOT NPPLOT
  /STATISTICS DESCRIPTIVES
  /CINTERVAL 95
  /MISSING LISTWISE
  /NOTOTAL.

* Levene's test for equality of variances included in T-TEST output above.

* ----- SECTION 8: GEOGRAPHIC PREFERENCE ANALYSIS -----

SORT CASES BY City.
SPLIT FILE SEPARATE BY City.

DESCRIPTIVES VARIABLES=MedIoT_Acceptance AIDecSupport_Acceptance
                        EnvDigHealth_Acceptance PrivacyConcern
  /STATISTICS=MEAN STDDEV MIN MAX.

SPLIT FILE OFF.

* ----- END OF SYNTAX -----
```

**Section 2: Data Dictionary (Selected Variables)**

| **Variable** | **Type** | **Label** | **Scale** | **Values/Notes** |
| --- | --- | --- | --- | --- |
| RespondentType | Nominal | Respondent group | — | 1=Elderly;  2=Family caregiver |
| City | Nominal | Geographic location | — | 1=Bangkok; 2=Chiang Mai |
| Age | Scale | Age in years | Continuous | Elderly: 60–88; Family: 35–55 |
| DigitalLiteracy | Ordinal | Self-rated digital literacy level | 1–5 | 1=None; 5=Expert |
| PU1–PU5 | Ordinal | Perceived Usefulness items 1–5 | 1–5 | 1=Strongly disagree; 5=Strongly agree |
| EOU1–EOU5 | Ordinal | Perceived Ease of Use items 1–5 | 1–5 | 1=Strongly disagree; 5=Strongly agree |
| SI1–SI4 | Ordinal | Social Influence items 1–4 | 1–5 | 1=Strongly disagree; 5=Strongly agree |
| FC1–FC4 | Ordinal | Facilitating Conditions items 1–4 | 1–5 | 1=Strongly disagree; 5=Strongly agree |
| BI1–BI3 | Ordinal | Behavioral Intention items 1–3 | 1–5 | 1=Strongly disagree; 5=Strongly agree |
| PU_Score | Scale | Composite Perceived Usefulness score (mean of PU1–5) | 1–5 | Computed variable |
| EOU_Score | Scale | Composite Ease of Use score (mean of EOU1–5) | 1–5 | Computed variable |
| SI_Score | Scale | Composite Social Influence score (mean of SI1–4) | 1–5 | Computed variable |
| FC_Score | Scale | Composite Facilitating Conditions score (mean of FC1–4) | 1–5 | Computed variable |
| BI_Score | Scale | Composite Behavioral Intention score (mean of BI1–3) | 1–5 | Computed variable |
| PrivacyConcern | Ordinal | Privacy concern rating | 1–5 | 1=No concern; 5=Extreme concern |
| TechVisibility | Ordinal | Preference for invisible vs. visible technology | 1–5 | 1=Strongly prefer visible; 5=Strongly prefer invisible |

Note. Anonymized dataset and full SPSS output files are available from the corresponding author upon request, subject to IRB conditions.
