# Supplementary material for: Gerontechnology Acceptance in Thai Older Adult Care Facilities—Sensor Network and Machine Learning Adoption: Mixed Methods Study
Source: JMIR Form Res. 2026 Aug 18;10:e91877. doi: 10.2196/91877 (PMC13484945; doi:10.2196/91877)
Supplement: Supplementary file 4 [file formative-v10-e91877-s004.docx]

**Multimedia Appendix 4**

**Qualitative Interview Guide and Representative Participant Quotations**

*Thai Original and English Translation*

Manuscript #91877 | JMIR Formative Research | Ongkasuwan M et al.

**PART A: Semi-Structured Interview Guide**

The following interview guide was developed iteratively, first based on the Phase 1 quantitative findings and then piloted with 3 participants (excluded from analysis).

The guide was administered in Thai by trained bilingual interviewers. Duration: 45–75 minutes. Audio-recorded with consent.

**Preamble / Introduction Script (Thai → English)**

**Thai:**	*"ขอบคุณที่สละเวลามาร่วมการสัมภาษณ์ในวันนี้ เราจะพูดคุยเกี่ยวกับความคิดเห็นและประสบการณ์ของท่านเกี่ยวกับการใช้เทคโนโลยีดิจิทัลในการดูแลผู้สูงอายุ ไม่มีคำตอบที่ถูกหรือผิด เราสนใจความคิดเห็นของท่านเอง การสัมภาษณ์จะใช้เวลาประมาณ 45–60 นาที"*

**English:**	*"*Thank you for taking the time to participate in today's interview. We will discuss your opinions and experiences regarding the use of digital technology in elderly care. There are no right or wrong answers; we are interested in your own views. The interview will take approximately 45–60 minutes."

**Section 1: General Technology Attitudes (Warm-Up)**

**Q1.1:**	*ท่านใช้โทรศัพท์มือถือหรืออุปกรณ์ดิจิทัลอื่นๆ บ้างไหม? ใช้ทำอะไร?*

**(EN)**	Do you use a mobile phone or other digital devices? What do you use them for?

**Q1.2:**	*ท่านรู้สึกอย่างไรกับเทคโนโลยีใหม่ๆ โดยทั่วไป?*

**(EN)**	How do you generally feel about new technology?

**Q1.3:**	*มีใครในครอบครัวหรือผู้ดูแลของท่านที่ช่วยท่านใช้เทคโนโลยีบ้างไหม?*

**(EN)**	Does anyone in your family or among your caregivers help you use technology?

**Section 2: Perceived Usefulness and Health Monitoring**

**Q2.1:**	*ถ้ามีเซ็นเซอร์ตรวจจับการหกล้มในห้องพักของท่าน ท่านคิดว่ามันจะมีประโยชน์แค่ไหน?*

**(EN)**	If there were a fall detection sensor in your room, how useful do you think it would be?

**Q2.2:**	*ท่านคิดว่าอุปกรณ์ที่วัดสัญญาณชีพ เช่น ชีพจรและออกซิเจนในเลือด จะช่วยให้ท่านมีสุขภาพดีขึ้นได้อย่างไร?*

**(EN)**	How do you think a device that measures vital signs like heart rate and blood oxygen could help you stay healthier?

**Q2.3:**	*สิ่งใดที่สำคัญที่สุดสำหรับท่านในการใช้ชีวิตอิสระในสถานดูแล?*

**(EN)**	What is most important to you about living independently in a care facility?

**Section 3: Privacy, Dignity, and Comfort**

**Q3.1:**	*ถ้ามีกล้องหรือเซ็นเซอร์คอยติดตามกิจกรรมของท่าน ท่านจะรู้สึกอย่างไร?*

**(EN)**	How would you feel if there were cameras or sensors monitoring your activities?

**Q3.2:**	*ท่านคิดว่าข้อมูลสุขภาพของท่านควรถูกเก็บและแชร์กับใครได้บ้าง?*

**(EN)**	Who do you think should have access to your health data?

**Q3.3:**	*มีประเภทของเทคโนโลยีใดที่ท่านจะรู้สึกไม่สบายใจถ้าต้องใช้ในการดูแลของท่าน?*

**(EN)**	Are there types of technology you would feel uncomfortable having in your care setting?

**Q3.4:**	*ระหว่างอุปกรณ์ที่สวมใส่กับเซ็นเซอร์ที่มองไม่เห็นในห้อง ท่านชอบแบบไหนมากกว่ากัน?*

**(EN)**	Between a wearable device and an invisible room sensor, which would you prefer?

**Section 4: Family Involvement and Social Influence**

**Q4.1:**	*ถ้าลูกหลานหรือผู้ดูแลแนะนำให้ท่านลองใช้เทคโนโลยีใหม่ ท่านจะทำตามคำแนะนำของพวกเขาไหม?*

**(EN)**	If your children or caregivers recommended trying a new technology, would you follow their advice?

**Q4.2:**	*ใครมีอิทธิพลมากที่สุดในการตัดสินใจว่าท่านจะใช้เทคโนโลยีอะไรในการดูแลของท่าน?*

**(EN)**	Who has the most influence over decisions about what technology you use in your care?

**Q4.3:**	*ท่านต้องการให้ครอบครัวสามารถดูข้อมูลสุขภาพของท่านผ่านแอปพลิเคชันได้ไหม?*

**(EN)**	Would you want your family to be able to see your health data through an application?

**Section 5: Ease of Use, Training, and Facilitating Conditions**

**Q5.1:**	*ท่านคิดว่าตัวเองจะต้องใช้เวลานานแค่ไหนในการเรียนรู้การใช้งานอุปกรณ์สุขภาพดิจิทัลใหม่?*

**(EN)**	How long do you think it would take you to learn to use a new digital health device?

**Q5.2:**	*ท่านต้องการความช่วยเหลือหรือการฝึกอบรมรูปแบบใดบ้างเพื่อให้รู้สึกมั่นใจในการใช้เทคโนโลยี?*

**(EN)**	What type of help or training would you need to feel confident using technology?

**Q5.3:**	*หากระบบเทคโนโลยีมีปัญหา ท่านอยากให้มีใครคอยช่วยเหลือท่านอย่างไร?*

**(EN)**	If the technology system had a problem, how would you want help to be provided?

**Section 6: Closing – Adoption Intention**

**Q6.1:**	*โดยรวมแล้ว ท่านมีความตั้งใจที่จะใช้เทคโนโลยีดิจิทัลเพื่อสุขภาพในสถานดูแลของท่านไหม?*

**(EN)**	Overall, do you intend to use digital health technology in your care facility?

**Q6.2:**	*มีอะไรที่จะทำให้ท่านมีแนวโน้มที่จะใช้เทคโนโลยีเหล่านี้มากขึ้นหรือน้อยลง?*

**(EN)**	What would make you more or less likely to use these technologies?

**Q6.3:**	*มีสิ่งอื่นใดที่เกี่ยวกับเทคโนโลยีดิจิทัลในการดูแลผู้สูงอายุที่ท่านอยากบอกเราไหม?*

**(EN)**	Is there anything else about digital technology in elderly care that you would like to tell us?

**PART B: Focus Group Guide**

The focus group guide followed a similar structure to the interview guide but was adapted for group discussion dynamics. Groups were 6–8 participants; duration 90 minutes. Thai language throughout. The following additional prompts were used to encourage group interaction.

**FG1:**	*"ผู้เข้าร่วมท่านอื่นคิดเห็นอย่างไรกับที่พูดไปเมื่อกี้?"*

**(EN)**	"What do other participants think about what was just said?"

**FG2:**	*"มีใครเคยมีประสบการณ์กับเทคโนโลยีสุขภาพมาก่อนบ้าง? ช่วยเล่าให้กลุ่มฟังได้ไหม?"*

**(EN)**	"Has anyone had experience with health technology before? Could you share that with the group?"

**FG3:**	*"คนในกรุงเทพฯ และในเชียงใหม่อาจมีความต้องการต่างกัน — มีใครอยากพูดถึงความแตกต่างเหล่านี้บ้าง?"*

**(EN)**	"People in Bangkok and Chiang Mai may have different needs — would anyone like to speak to these differences?"

**PART C: Representative Participant Quotations by Theme**

The following quotations are drawn from interview and focus group transcripts.

Thai originals are presented first, followed by English translation. All names are pseudonyms. CMI = Chiang Mai; BKK = Bangkok; E = Elderly participant; F = Family caregiver; S = Clinical staff.

**Theme 1: Surveillance Anxiety and Privacy as a Dignity Issue**

**Thai:**	*"ฉันไม่อยากให้ครอบครัวเห็นทุกครั้งที่ฉันเข้าห้องน้ำหรือนอนนานแค่ไหน ฉันยังคงเป็นคน ไม่ใช่ผู้ป่วยที่ต้องถูกสังเกต"*

**English:**	"I do not want my family to see every time I go to the bathroom or how long I sleep. I am still a person, not a patient to be observed."

*(Participant E-07, Female, 74 years, CMI)*

**Thai:**	*"เซ็นเซอร์ที่มองไม่เห็นนั้นโอเค แต่กล้องทำให้ฉันรู้สึกเหมือนอยู่ในโรงพยาบาล ฉันมาที่นี่เพื่อใช้ชีวิต ไม่ใช่เพื่อถูกดูแล"*

**English:**	"Invisible sensors are okay, but cameras make me feel like I am in a hospital. I came here to live, not to be watched."

*(Participant E-12, Male, 68 years, BKK)*

**Thai:**	*"ฉันเข้าใจว่าข้อมูลถูกเก็บเพื่อความปลอดภัยของฉัน แต่ฉันต้องการรู้ว่าใครสามารถดูได้บ้าง และฉันต้องการอำนาจในการตัดสินใจ"*

**English:**	"I understand the data is collected for my safety, but I need to know who can see it, and I need to have the power to decide."

*(Participant E-03, Female, 71 years, CMI)*

**Theme 2: Family-Mediated Technology Adoption**

**Thai:**	*"แม่ของฉันจะไม่ลองอะไรใหม่ๆ เลย ถ้าฉันไม่นั่งอยู่ด้วยและบอกว่ามันปลอดภัย ถ้าฉันเห็นด้วย แกก็ยอมรับ ถ้าฉันไม่อยู่ แกจะปฏิเสธ"*

**English:**	"My mother will not try anything new unless I sit beside her and tell her it is safe. If I approve, she accepts. If I am not there, she refuses."

*(Participant F-04, Female, 44 years, BKK)*

**Thai:**	*"ลูกๆ ของฉันรู้มากกว่าฉันเรื่องเทคโนโลยี ถ้าพวกเขาบอกว่ามันดี ฉันก็เชื่อพวกเขา นั่นคือวิธีที่ครอบครัวทำงาน"*

**English:**	"My children know more about technology than I do. If they say it is good, I trust them. That is how family works."

*(Participant E-15, Male, 79 years, CMI)*

**Thai:**	*"ในฐานะลูกคนโต ฉันรู้สึกว่าเป็นหน้าที่ของฉันในการตรวจสอบระบบทุกอย่างก่อนที่พ่อแม่จะตกลงที่จะใช้มัน"*

**English:**	"As the oldest child, I feel it is my duty to investigate every system before my parents agree to use it."

*(Participant F-09, Male, 51 years, BKK)*

**Theme 3: Regional Digital Trust and Cultural Moderation**

**Thai:**	*"เครื่องจักรไม่สามารถเข้าใจเรื่องบุญและกรรมได้ AI อาจพยากรณ์ร่างกาย แต่ไม่รู้จิตใจ การดูแลที่แท้จริงต้องมาจากคน"*

**English:**	"A machine cannot understand merit and karma. AI may predict the body, but it does not know the mind. True care must come from a person."

*(Participant E-18, Female, 82 years, CMI, Focus Group 3)*

**Thai:**	*"ในกรุงเทพฯ เราชินกับแอปพลิเคชันและการชำระเงินดิจิทัลแล้ว เทคโนโลยีสุขภาพก็แค่ขยายสิ่งที่เราทำอยู่แล้ว"*

**English:**	"In Bangkok, we are already used to applications and digital payments. Health technology is just an extension of what we already do."

*(Participant E-05, Male, 66 years, BKK)*

**Thai:**	*"ที่เชียงใหม่ เราไว้ใจคนที่รู้จักเรา ถ้าพยาบาลแนะนำเทคโนโลยี ฉันจะลอง แต่ถ้าเป็นแค่สัญญาณไฟจากเครื่อง ฉันไม่แน่ใจ"*

**English:**	"In Chiang Mai, we trust people who know us. If the nurse recommends the technology, I will try it. But if it is just a signal from a machine, I am not sure."

*(Participant E-20, Female, 73 years, CMI)*

**Theme 4: Training Needs and Caregiver Digital Literacy**

**Thai:**	*"ฉันกลัวที่จะทำพัง หรือกดผิดปุ่ม ถ้ามีคนมาสอนฉันทีละขั้นตอนและอยู่กับฉันจนกว่าฉันจะมั่นใจ ฉันก็คงจะลองได้"*

**English:**	"I am afraid of breaking something, or pressing the wrong button. If someone teaches me step by step and stays with me until I am confident, I think I could try."

*(Participant E-09, Female, 77 years, BKK)*

**Thai:**	*"เราต้องการการฝึกอบรมทุกสองสามเดือนเพราะซอฟต์แวร์มีการเปลี่ยนแปลง ถ้าเราไม่มั่นใจ เราก็ไม่สามารถช่วยให้ผู้สูงอายุมั่นใจได้"*

**English:**	"We need training every few months because the software changes. If we are not confident, we cannot help the residents be confident."

*(Nurse Coordinator, 38 years, BKK, Focus Group 1)*

**Thai:**	*"ลูกสาวของฉันต้องสอนฉันใหม่ทุกครั้งที่โทรศัพท์อัปเดต ฉันไม่ต้องการเทคโนโลยีที่เปลี่ยนแปลงตลอดเวลา"*

**English:**	"My daughter has to teach me again every time the phone updates. I do not want technology that keeps changing."

*(Participant E-11, Male, 80 years, CMI)*

**Theme 5: Dignity-Preserving Technology Design**

**Thai:**	*"ฉันอยากใช้ชีวิตเหมือนปกติ ไม่รู้สึกว่าอยู่ในโรงพยาบาล ถ้าเทคโนโลยีมองไม่เห็น ฉันก็ลืมได้ว่ามันอยู่ที่นั่น"*

**English:**	"I want to live like a normal person, not feel like I am in a hospital. If the technology is invisible, I can forget it is there."

*(Participant E-02, Female, 69 years, BKK)*

**Thai:**	*"อุปกรณ์สวมใส่ดูเหมือนโรงพยาบาล คนอื่นจะถามว่าฉันป่วยหนักหรือเปล่า เซ็นเซอร์ในห้องดีกว่ามาก"*

**English:**	"Wearable devices look medical. Other people will ask if I am seriously ill. Room sensors are much better."

*(Participant E-16, Female, 72 years, CMI)*

**Thai:**	*"เทคโนโลยีต้องรู้สึกเหมือนเพื่อน ไม่ใช่เครื่องมือการแพทย์ ถ้ามันทำให้ฉันรู้สึกเจ็บป่วย ฉันจะไม่ยอมรับมัน"*

**English:**	"Technology must feel like a companion, not a medical instrument. If it makes me feel sick, I will not accept it."

*(Participant E-08, Male, 75 years, BKK)*

Note. All quotations presented above are verbatim extracts from audio-recorded and transcribed interviews and focus groups. English translations were produced by bilingual research team members (MO and UC) and back-checked for accuracy. Original Thai transcripts are retained in the research archive.
