# Supplementary material for: Gerontechnology Acceptance in Thai Older Adult Care Facilities—Sensor Network and Machine Learning Adoption: Mixed Methods Study
Source: JMIR Form Res. 2026 Aug 18;10:e91877. doi: 10.2196/91877 (PMC13484945; doi:10.2196/91877)
Supplement: Supplementary file 5 [file formative-v10-e91877-s005.docx]

**Multimedia Appendix 1**

**STROBE Validation Checklist**

*Strengthening the* ***R****eporting of* ***Ob****servational Studies in* ***E****pidemiology in Healthcare Services*

Manuscript #91877 | JMIR Formative Research | Ongkasuwan M et al.

The STROBE (Strengthening the Reporting of Observational Studies in Epidemiology) for healthcare Service checklist is used to validate the reporting of the cross-sectional survey components of this mixed-methods study.

Items are mapped from ***reviewers' recommendations*** to page/section locations in the revised manuscript Number 91877.

| **Section/Topic** | **Item #** | **Reviewers’ Recommendation** | **Reported on Page / Section for revision** |
| --- | --- | --- | --- |
| Title & Abstract | 1a | Indicate the study's design with a commonly used term in the title or the abstract | Title page; Abstract |
|  | 1b | Provide in the abstract an informative and balanced summary of what was done and what was found | Abstract (pp.1–2) |
| **Introduction** | | | |
| Background / Rationale | 2 | Explain the scientific background and rationale for the investigation being reported | Introduction, paragraphs 1–2 (p.3) |
| Objectives | 3 | State-specific objectives, including any prespecified hypotheses | Introduction: Study Objectives (p.4) |
| **Methods** | | | |
| Study Design | 4 | Present key elements of the study design early in the paper | Methods: Study Design (p.5) |
| Setting | 5 | Describe the setting, locations, and relevant dates, including periods of recruitment, exposure, follow-up, and data collection | Methods: Participants and Sampling (p.6) |
| Participants | 6a | Cohort study – Provide the eligibility criteria and the sources and methods used to select participants. Describe methods of follow-up | Methods: Participants and Sampling (p.6) |
|  | 6b | Cross-sectional study – Give the eligibility criteria, and the sources and methods of selection of participants | Methods: Participants and Sampling (p.6) |
| Variables | 7 | Clearly define all outcomes, exposures, predictors, potential confounders, and effect modifiers. Give diagnostic criteria, if applicable | Methods: Instrumentation; GTAM Mapping Table (p.7) |
| Data Sources / Measurement | 8 | For each variable of interest, give sources of data and details of the methods of assessment. Describe the comparability of assessment methods if there is more than one group | Methods: Instrumentation (pp.7–8) |
| Bias | 9 | Describe any efforts to address potential sources of bias | Methods & Discussion: Limitations (p.9, p.18) |
| Study Size | 10 | Explain how the study size was arrived at | Methods: Participants (p.6) — purposive sampling rationale given |
| Quantitative Variables | 11 | Explain how quantitative variables were handled in the analyses. If applicable, describe which groupings were chosen and why | Methods: Data Analysis (p.9) |
| Statistical Methods | 12a | Describe all statistical methods, including those used to control for confounding | Methods: Data Analysis (p.9) |
|  | 12b | Describe any methods used to examine subgroups and interactions | Methods: Data Analysis — geographic subgroup analysis (p.9) |
|  | 12c | Explain how the missing data were addressed | Methods: Data Analysis — complete case analysis (p.9) |
|  | 12d | Cross-sectional study – If applicable, describe analytical methods taking account of sampling strategy | Methods: Data Analysis (p.9) |
|  | 12e | Describe any sensitivity analyses | Not applicable for this study design |
| **Results** | | | |
| Participants | 13a | Report numbers of individuals at each stage of study — e.g., numbers potentially eligible, examined for eligibility, confirmed eligible, included in the study, completing follow-up, and analyzed | Methods: Participants (p.6); Results: Table 3 (p.11) |
|  | 13b | Give reasons for non-participation at each stage | Methods: Participants — no significant non-participation noted (p.6) |
|  | 13c | Consider the use of a flow diagram | Not included (simple two-group recruitment); added to revision if required |
| Descriptive Data | 14a | Give characteristics of study participants (e.g., demographic, clinical, social) and information on exposures and potential confounders | Results: Participants described (p.10); Tables 1–3 |
|  | 14b | Indicate the number of participants with missing data for each variable of interest | Results — complete data for all 120 consumer participants and 12 specialists; no missing data reported |
|  | 14c | Cohort study – Summarize follow-up time (e.g., average and total amount) | N/A — cross-sectional design |
| Outcome Data | 15 | Report the number of outcome events or summary measures | Results: Tables 1–4 (pp.10–14) |
| Main Results | 16a | Give unadjusted estimates and, if applicable, confounder-adjusted estimates and their precision (e.g., 95% CI). Make clear which confounders were adjusted for and why they were included | Results: Table 3; regression results (p.13) |
|  | 16b | Report category boundaries when continuous variables were categorized | Results: Digital literacy dichotomization described (p.12) |
|  | 16c | If relevant, consider translating estimates of relative risk into absolute risk for a meaningful time period | N/A — cross-sectional acceptance study |
| Other Analyses | 17 | Report other analyses done — e.g., analyses of subgroups and interactions, and sensitivity analyses | Results: Geographic subgroup analysis (p.14) |
| **Discussion** | | | |
| Key Results | 18 | Summarize key results with reference to study objectives | Discussion: Principal Findings (p.16) |
| Limitations | 19 | Discuss limitations of the study, taking into account sources of potential bias or imprecision. Discuss both the direction and the magnitude of any potential bias | Discussion: Limitations (p.18) |
| Interpretation | 20 | Give a cautious overall interpretation of results, considering objectives, limitations, multiplicity of analyses, results from similar studies, and other relevant evidence | Discussion (pp.16–19) |
| Generalizability | 21 | Discuss the generalizability (external validity) of the study results | Discussion: Scalability and Generalizability (p.18) |
| **Other** | | | |
| Funding | 22 | Give the source of funding and the role of the funders for the present study and, if applicable, for the original study on which the present article is based | Funding Statement (p.20) |

Note. STROBE Validation Statement checklist adapted from: von Elm E, Altman DG, Egger M, Pocock SJ, Gøtzsche PC, Vandenbroucke JP; STROBE Initiative. Strengthening the reporting of observational studies in epidemiology or health service (STROBE) statement: guidelines for reporting observational studies. BMJ. 2007;335(7624):806-808. doi:10.1136/bmj.39335.541782.AD
