# Supplementary material for: Gerontechnology Acceptance in Thai Older Adult Care Facilities—Sensor Network and Machine Learning Adoption: Mixed Methods Study
Source: JMIR Form Res. 2026 Aug 18;10:e91877. doi: 10.2196/91877 (PMC13484945; doi:10.2196/91877)
Supplement: Supplementary file 6 [file formative-v10-e91877-s006.docx]

**Multimedia Appendix 2**

**COREQ for Qualitative Research Checklist**

*Consolidated Criteria for Reporting Qualitative Research*

Manuscript #91877 | JMIR Formative Research | Ongkasuwan M et al.

The **COREQ** (**Co**nsolidated Criteria for **Re**porting **Q**ualitative Research) 32-item checklist is completed below for the **qualitative component** (Phase 2 of this study) of this sequential explanatory mixed-methods study, covering semi-structured interviews (n=20) and focus groups (n=3).

| **Domain / Topic** | **No.** | **Item** | **Guiding Question** | **Response / Location in Manuscript** |
| --- | --- | --- | --- | --- |
| **DOMAIN 1: RESEARCH TEAM AND REFLEXIVITY** | | | | |
| Personal Characteristics | 1 | Interviewer/facilitator | Which author(s) conducted the interview or focus group? | MO and UC conducted all semi-structured interviews.  MO and AJ facilitated the three focus groups |
|  | 2 | Credentials | What were the researcher's credentials, e.g., a PhD or an MD? | MO: PhD (Associate Professor);  UC: PhD (Assistant Professor);  AJ: PhD (Lecturer);  PS: PhD (Lecturer) |
|  | 3 | Occupation | What was their occupation at the time of the study? | All authors: Academic researchers, MSME School of Management and Economics, Assumption University |
|  | 4 | Gender | Was the researcher male or female? | MO: Female;  UC: Female;  AJ: Male;  PS: Female |
|  | 5 | Experience and training | What experience or training did the researcher have? | MO and UC have >10 years of qualitative research experience in gerontechnology and health services; trained in thematic analysis (Braun & Clarke method) |
| Relationship with participants | 6 | Relationship established | Was a relationship established prior to study commencement? | No prior relationship with participants; first contact occurred during recruitment through community health centers |
|  | 7 | Participant's knowledge of the interviewer | What did the participants know about the researcher, e.g., personal goals, reasons for doing the research? | Participants were informed that the research team consisted of university academics conducting a study on technology acceptance in elderly care.  institutional affiliation was disclosed |
|  | 8 | Interviewer characteristics | What characteristics were reported about the interviewer/facilitator? | Female lead interviewer (MO) experienced in elderly health technology research;  bilingual Thai-English;  familiar with local care settings |
| Theoretical Framework | 9 | Methodological orientation and theory | What methodological orientation was stated to underpin the study? | Thematic analysis following Braun & Clarke (2006), grounded in GTAM to explain quantitative adoption patterns through qualitative data |
|  | 10 | Reflexivity | What consideration was given to the researcher's reflexivity? | Researchers maintained reflective journals during data collection and analysis phases.  Regular team debriefs were held to surface assumptions and manage potential confirmation bias, given researchers' prior quantitative work in the same domain |
| **DOMAIN 2: STUDY DESIGN** | | | | |
| Participant Selection | 11 | Sampling | How were participants selected (e.g., purposive, convenience, consecutive, snowball)? | Purposive sampling from Phase 1 survey participants; selected to maximize variation in age, digital literacy level, and geographic location |
|  | 12 | Method of approach | How were participants approached (e.g., face-to-face, telephone, mail, or email)? | Face-to-face approach at community health centers; Phase 1 participants re-contacted by bilingual research assistant via telephone |
|  | 13 | Sample size | How many participants were in the study? | Semi-structured interviews: n=20 (12 elderly adults, 8 adult family members); Focus groups: 3 groups, 6–8 participants each (total n=22 focus group participants) |
|  | 14 | Non-participation | How many people refused to participate or dropped out? Reasons? | 3 initially consenting Phase 1 participants declined interview due to scheduling conflicts; 1 withdrew during focus group (fatigue). No replacements were sought, as saturation had been reached. |
| Setting | 15 | Setting of data collection | Where was the data collected (e.g., home, clinic, workplace)? | Community health centers and senior activity centers in Bangkok (n=2 sites) and Chiang Mai (n=2 sites) |
|  | 16 | Presence of non-participants | Was anyone else present besides the participants and researchers? | Family members were permitted to sit nearby (but not in the interview space) per participant request, in 4 cases; they did not participate in or observe the interview |
|  | 17 | Description of sample | What are the important characteristics of the sample, e.g., demographic data, date? | See the table below; data collected March–June 2024 |
| Data Collection | 18 | Interview guide | Were questions, prompts, or guides provided by the authors? Was it pilot tested? | A semi-structured interview guide was developed based on Phase 1 quantitative findings. Piloted with 3 participants (not included in analysis); refined for clarity and cultural appropriateness.  See full guide in Appendix 6. |
|  | 19 | Repeat interviews | Were repeat interviews carried out? If yes, how many? | No repeat interviews conducted; single session per participant (45–75 min for interviews, 90 min for focus groups) |
|  | 20 | Audio/visual recording | Did the research use audio or visual recording to collect the data? | All interviews and focus groups were audio-recorded with participant consent; transcribed verbatim in Thai |
|  | 21 | Field notes | Were field notes made during and/or after the interview or focus group? | Field notes recorded after each session by the interviewing researcher; reviewed during thematic analysis |
| **DOMAIN 3: ANALYSIS AND FINDINGS** | | | | |
| Data Analysis | 22 | Data analysis | How were codes derived? Was data analysis done by more than one researcher? | Thematic analysis per Braun & Clarke (2006); initial codes derived inductively from data; two researchers (MO, UC) coded independently; inter-coder reliability assessed (Cohen's κ=0.78);  disagreements resolved by consensus discussion |
|  | 23 | Description of the coding tree | Did the authors provide sufficient detail to replicate the coding? | Codebook is available as part of this appendix (Section 3 below) |
|  | 24 | Derivation of themes | Were themes identified in advance or derived from the data? | Themes derived from the data (inductive); subsequently interpreted in relation to GTAM constructs (deductive phase) |
|  | 25 | Software | What software, if applicable, was used to manage the data? | NVivo 14 (QSR International) is used for data management, coding, and theme mapping |
|  | 26 | Participant checking | Did participants provide feedback on the findings? | Member checking conducted with 6 participants (3 interviews, 3 focus groups) via telephone; minor clarifications incorporated |
| Reporting | 27 | Quotations presented | Were participant quotations presented to illustrate the themes/findings? | Yes, representative quotations are presented in the Results section of the main manuscript, and the full set is in Multimedia Appendix 6 |
|  | 28 | Data and findings consistent | Was there consistency between the data presented and the findings? | Yes, all quotations presented are directly from transcripts and mapped to specific coded extracts verified by two researchers |
|  | 29 | Clarity of major themes | Were major themes clearly presented in the findings? | Yes, five themes are presented with definitions, analytic commentary, and supporting quotations in the Results section |
|  | 30 | Clarity of minor themes | Is there a description of diverse cases or a discussion of minor themes? | Minor sub-themes noted within each major theme; full codebook and theme map available in this appendix |

**Section 2: Qualitative Participant Characteristics**

The following table summarises the characteristics of the 20 interview and focus group participants.

| **Characteristic** | **Elderly – Category A** | **n** | **Elderly – Category B** | **n** | **Family – Category A** | **n** | **Family – n** |
| --- | --- | --- | --- | --- | --- | --- | --- |
| Age group | 60–69 years | 12 | 70–79 years | 7 | 35–45 years | 10 | 45–55 years |
| Gender | Female | 7 | Male | 5 | Female | 8 | Male |
| Location | Bangkok | 10 | Chiang Mai | 10 | Bangkok | 10 | Chiang Mai |
| Digital literacy | High | 6 | Low-medium | 6 | High | 10 | Medium |
| Education | University | 5 | Secondary or below | 7 | University | 10 | Secondary |

**Section 3: Abbreviated Codebook**

The following presents the primary codes associated with each of the five major themes identified in Phase 2. Full NVivo codebook available upon request.

| **Theme** | **Primary Codes** | **Linked GTAM Construct** |
| --- | --- | --- |
| Theme 1:  Surveillance Anxiety | camera_fear; data_sharing_concern; body_exposure_discomfort; family_data_access; hospital_feeling | Perceived Ease of Use; Social Influence |
| Theme 2:  Family-Mediated Adoption | family_endorsement; caregiver_as_gatekeeper; shared_decision_making; family_present_consent; trust_through_family | Social Influence (expanded gatekeeper role) |
| Theme 3:  Regional Digital Trust | BKK_tech_familiarity; CMI_face_to_face_preference; AI_mistrust_karma; urban_rural_digital_gap; connectivity_confidence | Facilitating Conditions: Social Influence |
| Theme 4:  Training Needs | fear_of_breaking; software_update_anxiety; staff_training_frequency; caregiver_literacy; step_by_step_learning | Facilitating Conditions: Perceived Ease of Use |
| Theme 5:  Dignity-Preserving Design | invisible_tech_preference; wearable_rejection; hospital_stigma_avoidance; ambient_monitoring_ok; person_not_patient | Perceived Usefulness; Behavioral Intention |

Note. COREQ checklist adapted from: Tong A, Sainsbury P, Craig J. Consolidated criteria for reporting qualitative research (COREQ): a 32-item checklist for interviews and focus groups. Int J Qual Health Care. 2007;19(6):349-357. doi:10.1093/intqhc/mzm042
